# [Expression of Concern] Platelet-rich plasma shows beneficial effects for patients with knee osteoarthritis by suppressing inflammatory factors

**DOI:** 10.3892/etm.2026.13269

**Published:** 2026-08-12

**Authors:** Guilin Huang, Sha Hua, Tuanmin Yang, Jianbing Ma, Wenxing Yu, Xiujin Chen

Exp Ther Med 15:3096–3102, 2018; DOI: 10.3892/etm.2018.5794

Following the publication of this paper, a number of concerns were brought to the attention of the Editorial Office by a concerned reader. First, it was noted that the article did not provide details of the name of the institution granting the ethics approval, the ethics approval number, or the date. In addition, the following comments/concerns were raised: First, the article states that 366 people aged 18-30 years with knee osteoarthritis were recruited, although, given that the prevalence of osteoarthritis in people under 30 years of age is very low, this appears to have been a surprisingly large number of people to have recruited for the study. It was also noted that the article did not provide a CONSORT flow diagram. The reader also observed that the article reports that participants were randomly divided into two groups, although multiple groups are presented in the results; moreover, in Fig. 1A and B, there are multiple placebo groups shown, but the text only mentions one placebo group. Finally, other omissions in the description of the methods were noted: The article did not report on either the method of generating the random sequence or the method for concealing the randomization sequence from both participants and researchers, and neither was the cut-off used to define the response rate provided for the Karnofsky scale.

The authors have been contacted by the Editorial Office to offer an explanation for the apparent anomalies and omissions in this paper, and we are awaiting their response. Owing to the fact that the Editorial Office has been made aware that important information has not been reported in this paper, we are issuing an Expression of Concern to notify readers of this potential problem while the Editorial Office continues to investigate this matter further.

